# Robinin inhibits pancreatic cancer cell proliferation, EMT and inflammation via regulating TLR2-PI3k-AKT signaling pathway

**DOI:** 10.1186/s12935-023-03167-3

**Published:** 2023-12-18

**Authors:** Wenwen Zhang, Wenting Liu, Xingchen Hu

**Affiliations:** 1 Department of Hernia Surgery, The Second People’s Hospital of Changzhou Affiliated to Nanjing Medical University, Changzhou, Jiangsu China; 2 Department of Ophthalmology, Huadong Hospital, Fudan University, Shanghai, China; 3 Present Address: Department of Burn Surgery, The Second People’s Hospital of Changzhou Affiliated to Nanjing Medical University, Changzhou, Jiangsu 213003 China

**Keywords:** Robinin, Pancreatic cancer, TLR2, Inflammation, Tumour microenvironment

## Abstract

**Purpose:**

To investigate the anti-tumor effect of Robinin (Toll-like receptor 2 inhibitor) in pancreatic cancer cells via regulating tumor microenvironment.

**Methods:**

The effects of Robinin on cell proliferation or migration in Mia-PACA2 and PANC-1 were determined, using CCK8 or wound healing assay, respectively. The typical markers of EMT (αSMA and snail) and the inflammation markers (IL-6 and TNF-α) were all detected by western blot. CU-T12-9 (TLR2 agonist) was used to rescue Robinin’s effect. PI3k-p85α and Phosphorylated-AKT (p-AKT) were evaluated, compared to the β-actin and AKT, using western blot.

**Results:**

Robinin significantly inhibited cell proliferation and migration in Mia-PACA2 and PANC-1, compared to HPNE (**P < 0.01). Robinin also attenuated the expression of α-SMA and snail in Mia-PACA2, and PANC-1 (**P < 0.01). Besides, it was found that expression of IL-6 and TNF-α were diminished in presence of Robinin in Mia-PACA2, and PANC-1 (**P < 0.01). Western blot confirmed that Robinin could target on TLR2, and further downregulated PI3k-AKT signaling pathway to exert biological function.

**Conclusions:**

Robinin exerts anti-tumor effect perhaps *via* downregulating inflammation and EMT in pancreatic cancer cell through inhibiting TLR2-PI3k-AKT signaling pathway. Robinin may be a novel agent in adjuvant therapy of pancreatic cancer.

**Supplementary Information:**

The online version contains supplementary material available at 10.1186/s12935-023-03167-3.

## Introduction

Pancreatic cancer (PC) has projected as the second leading cause of cancer death by the year 2020, which kills at least 200,000 people worldwide each year [1]. Once pancreatic cancer patients develop distant metastasis, the median survival drops to about 6 months, even with the most advanced techniques treatment [2]. Early radical operation is the only effective treatment for PC. However, lack of detection means for early diagnosis of pancreatic cancer, most patients miss the chance to resect tumor completely. Only 15% of patients with PC have the chance to undergo complete resection, while the remaining 85% of PC patients can only treated with either systemic chemotherapy or radiotherapy [3]. Chemotherapy combined with immune checkpoint blocking therapy has been very successful in a variety of cancers, including breast, lung and stomach cancers, but the chemotherapy is still not ideal in pancreatic cancer [4, 5]. Thus, to explore new anti-tumor therapy for patients who are unable to perform surgery possess great clinical value.

As reported in the literature, Toll-like receptors (TLRs) are a conserved receptor family, widely recognized for their ability to respond to pathogenic structures [6]. TLRS are widely distributed in a variety of immune cells and tumor-associated macrophages(TAMs) [7, 8]. Studies have shown that TLRs signaling pathway has been proven to be associated with the occurrence and prognosis of various cancers [9]. Based on differential expression analysis of pancreatic cancer tissues and surrounding tissues, most TLR genes are usually upregulated or downregulated to varying degrees in different cancer tissues. In pancreatic cancer, the expression of TLR2 and TLR4 is mainly increased, and is associated with higher mortality [10],while TLR9 shows a downward trend in pancreatic cancer [11]. Other molecules of the TLR family (TLR3,7,8) were also confirmed to be closely related to occurrence, development and invasion of pancreatic cancer [12, 13]. Therefore, we speculated that TLR2 interference can similarly regulate tumor microenvironment so as to minimize pan-cancer cell growth and attenuate metastasis, which would be a good candidate of pancreatic cancer medical therapy.

**Fig. 1:**
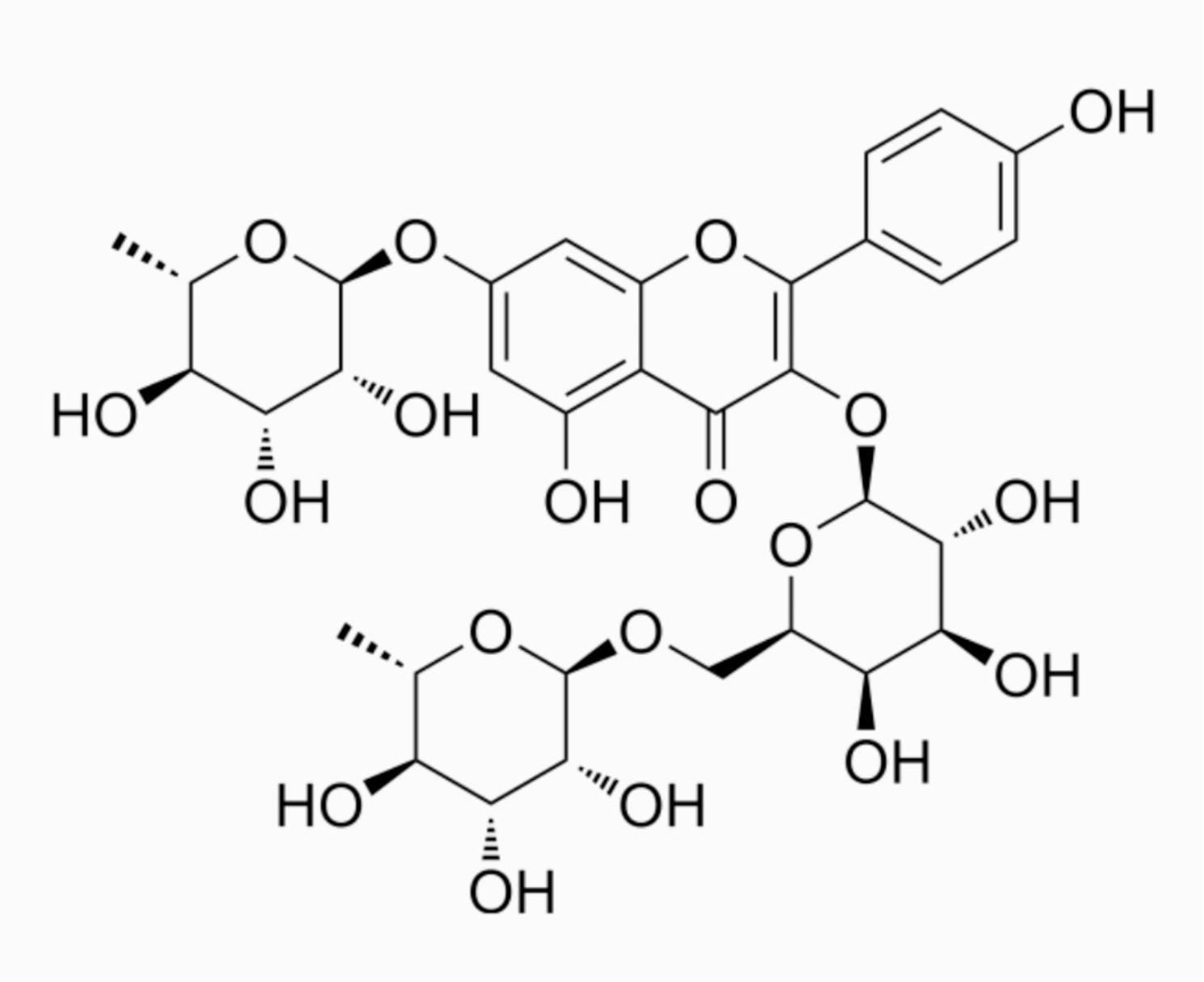
Chemical structure of Robinin

Robinin, a compound with molecular weight of 740.66Da, is present in flavonoid fraction of Vigna unguiculata leaf [14]. Its chemical structure is shown in Fig. 1. Robinin can inhibit upregulated expression of TLR2 and TLR4 to alleviate inflammatory and immune response [15]. Considering the high expression of TLR2 in pancreatic cancer and higher mortality, we speculated that Robinin (TLR2inhibitor) may play a positive role in pancreatic cancer therapy. However, the association between TLR2 stimulation and tumor-genesis of pancreatic cancer and underlying mechanism need to be further elucidated.

The aim of this study was to investigate anti-tumor effect of Robinin in Mia-PACA2 and PANC-1 in vitro. The underlying mechanism was also investigated to illustrate the possible signal pathway which acted by Robinin. These findings may aid to develop Robinin as a novel adjuvant therapy for pancreatic cancer in clinical.

## Materials and methods

### Cell culture

One normal human pancreatic ductal epithelial cell line HPNE (Shanghai Fuheng Biotechnology Co., Ltd., Shanghai, China) and two PC cell lines Mia-PACA2, and PANC-1 (ATCC, Manassas, VA, USA; www.atcc. org) were sourced from ATCC. One normal human pancreatic ductal epithelial cell line HPNE(Shanghai Fuhsinuo Technology Co., LTD., Shanghai, China) and two PC cell lines Mia-PACA2 and PANC-1 (ATCC, Manassas, VA, USA); www.Writeatcc. org) from ATCC. Cell lines were treated with 10% fetal bovine serum (FBS, Gibco Life Technologies) and 1%100 U/mL penicillin and 100 g/mL streptomycin (Gibco Life Technologies) in Dulbecco’s modified Eagle medium with high sugar content. The cells were incubated in an incubator containing 5% CO2 at 37℃, and the medium was changed every 2 days.

### Cytotoxicity and cell proliferation assay

Cytotoxicity assay was detected, using Cell Counting kit‑8 (CCK‑8; Dojindo Molecular Technologies, Inc., Kumamoto, Japan) according to the manufacturer’s instructions. The Cells were inoculated in 96-well plates according to the cell density of 1 × 104 Cells /well, and cultured in FBs-free medium for 24 h. After pre-starvation, incubated with 500 nM, 1 µM, 5 µM and 10 µM/ml Robinin (Robinin was purchased from MCE, according to Robinin’s instruction manual, Robinin in powder state was dissolved with DMSO) for 24 h in an incubator at 37℃. 24 h later, all culture-medium in 96-well plates were discarded and there was no time to add 10 µl CCK-8 solution, and incubated at 37℃ for 2 h. Colorimetric absorbance at 450 nm was recorded using a Bio-Rad Laboratories, Inc., Hercules, CA, USA.

The cells were pretreated with 1 µM/ml of Robinin and incubated with 10 µl of CCK8 for 1.5 h. The colorimetric absorbance of the cells at 450 nm was detected at 24, 48 and 72 h, respectively. The proliferation of Mia PaCa2 and PANC-1 cells was determined. In addition, CU-T12-9 was selectively added to reverse the effect of Robinin, and the proliferation of cells was detected by the same method described above. Finally, the multiplication curve is drawn.

### Wound healing assay

Mia-PACA2 was incubated with PANC-1 until 100% confluxed. A straight artificial wound was made with the tip of a 200 µl pipet. The scratch area of the cells at 0 h was imaged with a phase contrast microscope (magnification, x4). The cells were cultured with fresh serum-free medium or serum-free medium combined with Robinin (1 µM/ml) for 24 h, and CU-T12-9 was selectively added to reverse the effect of Robinin. After different treatments, the scratch areas of 0 and 24 h cells were photographed with phase contrast microscope (magnification, x4), and the cell migration distance was measured with Image j.

### RNA isolation, quantitative real-time PCR (qRT-PCR)

Trizol reagent (Invitrogen, USA) was used to extract total RNA from cells according to manufacturer’s instructions. For qRT-PCR, 2 µg total RNA was treated with DNaseI and reverse transcription was performed using the MMLV system (Promega, USA). ABI 7900 RT-PCR system was used for RT-PCR, and SYBR Green Real-time PCR Master Mix from American ABI was used for fluorescence quantitation, with GAPDH as the internal reference. mRNA abundance relative to GAPDH expression was calculated by 2−∆ Ct. Details of primers are shown in Table 1. The expression of TLR2, α-SMA, snail, IL-6 and TNF-α in HPNE, mia-paca2 and panc-1 were detected by RT-qPCR.

**Table 1:** Weight loss and tumor volume between control and Robinin treatment group

Name	Primer sequences (5’–3’)
TLR2	Forward: ATCCTCCAATCAGGCTTCTCT Reverse: GGACAGGTCAAGGCTTTTTACA
Snail	Forward: GCCGAGTGTAATCAGGAG
	Reverse: GGAGTGCTTGTTCAGGAG
α-SMA	Forward: CTTGAGAAGAGTTACGAGTTG
	Reverse: GATGCTGTTGTAGGTGGTT
IL-6	Forward: AGACAGCCACTCACCTCTTCA Reverse: GGCTTGTTCCTCACTACTCTC
TNF-α	Forward: CAGCCTTCATCCACTCTC Reverse: CATCTCTTGCCACATCTCT

### Western blot

RIPA lysis buffer (Thermo Fisher Scientific, Inc.), phosphatase inhibitor (Thermo Fisher Scientific, Inc.) and protease inhibitor (Thermo Fisher Scientific, Inc., Inc. to extract samples of total proteins from cells. BCA protein kit (Beyotime, Inc.) was used to detect protein concentration to make protein standard curve, and then loading buffer of corresponding volume was added into boiling water for 10 min to prepare protein samples. Protein samples (10 µg) were separated by SDS-PAGE with 10% separation gel and 5% stack gel and transferred to a polyvinylidene fluoride membrane (PVDF membrane, Millipore Corp., Bedford, MA, USA). Seal at room temperature with 5% skim milk powder for 2 h, then incubate at 4℃ overnight with primary antibody diluent. On day 2, tris buffered brine was washed 3 times with 0.1% Tween-20 detergent, and incubated with secondary antibody diluent at room temperature for 2 h. Finally, bands of immune response were visualized using enhanced chemiluminescence (Beyotime, Inc.) and protein bands were captured by a GS-700 imaging densitometer (BIO-RAD, Hercules, CA, USA). Finally, Image J was used to analyze the protein bands.

### Methods and materials

#### Pancreatic cancer induction and robinin intervention

Mouse experiments were performed following National Institutes of Health (NIH) guidelines. All animal studies complied with relevant ethical regulations for animal testing and research, and were approved by the Institutional Animal Care and Use Committee of the Second Changzhou Hospital. Six female mice (NKG) at 8 weeks old were used in all experiments. The subcutaneous and orthotopic pancreatic cancer model were established by subcutaneous incubation of 6 × 106 Panc-1 cells into the left back and the parenchyma of the pancreas of 8-week-old NKG mice, respectively. After 2 weeks, palpable tumors had developed whose diameters researched 7–8 mm. After model mice were successfully inducted, Robinin intervention was conducted. Gastric irrigation of Robinin (50 mg/Kg) was applied for treatment, while PBS was applied for control group.

### Analyses of tumor volume and weight loss

Pancreatic cancer development and progression was characterized by mice weight loss and tumor volume. The weights of mice were measured twice a week to calculate weight loss and plot weight curves. After 3 weeks intervention, mice were euthanized and tumor tissues were dissected. Tumor tissues were weighted and calculated by following formula: tumor volume (mm3) = 1/2 × a × b2 (a: the major diameter and b: the minor diameter). Finally, tumor tissues were fixed in 4% paraformaldehyde for 24 h, washed, embedded in paraffin, and sectioned at 10 µM for immunohistochemistry.

### Immunohistochemistry (IHC)

Tumor Sect. (4 μm) was dewaxed in xylene, hydrated in decreasing concentrations for 30 min, washed with phosphate-buffered saline, and probed with monoclonal antibodies or isotype controls at 4 °C overnight. The primary antibodies were used in this concentration (α-SMA: 1:300, vimentin-1:1:500). After being washed, the sections were incubated with biotinylated goat anti-rabbit or anti-mouse IgG for two hours at room temperature. Finally, the explants were embedded in the Tissue compound (Sakura Finetek Europe, Alphen, the Netherlands) and cut into 12 μm sections on a cryostat (CM1900; Leica Microsystems, Wetzlar, Germany). Tissue sections were collected onto microscope slides, air-dried, and stored at − 20 °C until use. Hematoxylin was used to visualize the nuclei. Finally, the samples were mounted in fluorescent mounting medium and cover slipped. All experiments were conducted in triplicate. Immunostaining was visualized with streptavidin/peroxidase complex and diaminobenzidine, and sections were counterstained with hematoxylin. Slides were visualized under a bright-field microscope at ×2.5 and ×5 magnification. Immunofluorescence staining images were taken by ZEISS microscope (LSM880, Germany). Positive cells and positive rate were quantified using Image-pro plus 6.0 (Media Cybernetics, Inc., Rockville, MD, USA) and expressed as mean ± SEM in high-powered fields detected by microscopy.

### Statistical analysis

Data were expressed as mean ± standard deviation (SD) using SPSS V17.0 software (SPSS Inc.; GraphPad Prism version 7.0 (GraphPad Software, Inc., La Jolla, CA, USA) for statistical analysis. Independent T test ((LSD) post hoc test) was used between the two groups, and univariate analysis of variance (ANOVA) was used to compare the multiple groups. Results were expressed as P values, where *P < 0.05 was considered statistically significant.

## Results

### Upregulated of TLR2, EMT and inflammation are determined in mia-PACA2 and PANC-1

**Fig. 2:**
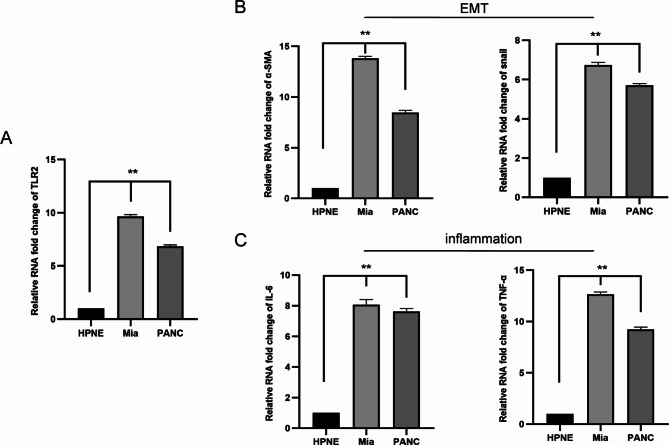
Expression levels of TLR2, EMT, and inflammation markers in HPNE, Mia-PACA2 and PANC-1. **(A)** qRT-PCR of TLR2 in HPNE, Mia-PACA2 and PANC-1. **(B)** qRT-PCR of α-SMA and snail expression in HPNE, Mia-PACA2 and PANC-1. **(C)** qRT-PCR of IL-6 and TNF-α expression in HPNE, Mia-PACA2 and PANC-1, Mia-PACA2 and PANC-1 vs. HPNE cell line, *p < 0.05, ** *p* < 0.01. Measurement data were expressed as mean ± standard derivation; data between PC cell and HPNE were compared by t-test, and measurement data among multiple groups were analyzed by one-way analysis of variance with Tukey’s post hoc test. The experiment was repeated three times

Compared with normal HPNE, TLR2 was found highly expressed in Mia-PACA2 and PANC-1 (**P < 0.01, Fig. 2A). Snail and α-SMA also highly expressed in Mia-PACA2 and PANC-1, which indicated that EMT was active in pancreatic cancer cells (**P < 0.01, Fig. 2B). Furthermore, inflammation markers (IL-6 and TNF-α) are highly expressed in Mia-PACA2 and PANC-1 (**P < 0.01, Fig. 2C).

### Robinin attenuates cell proliferation of mia-PACA2 and PANC-1

**Fig. 3:**
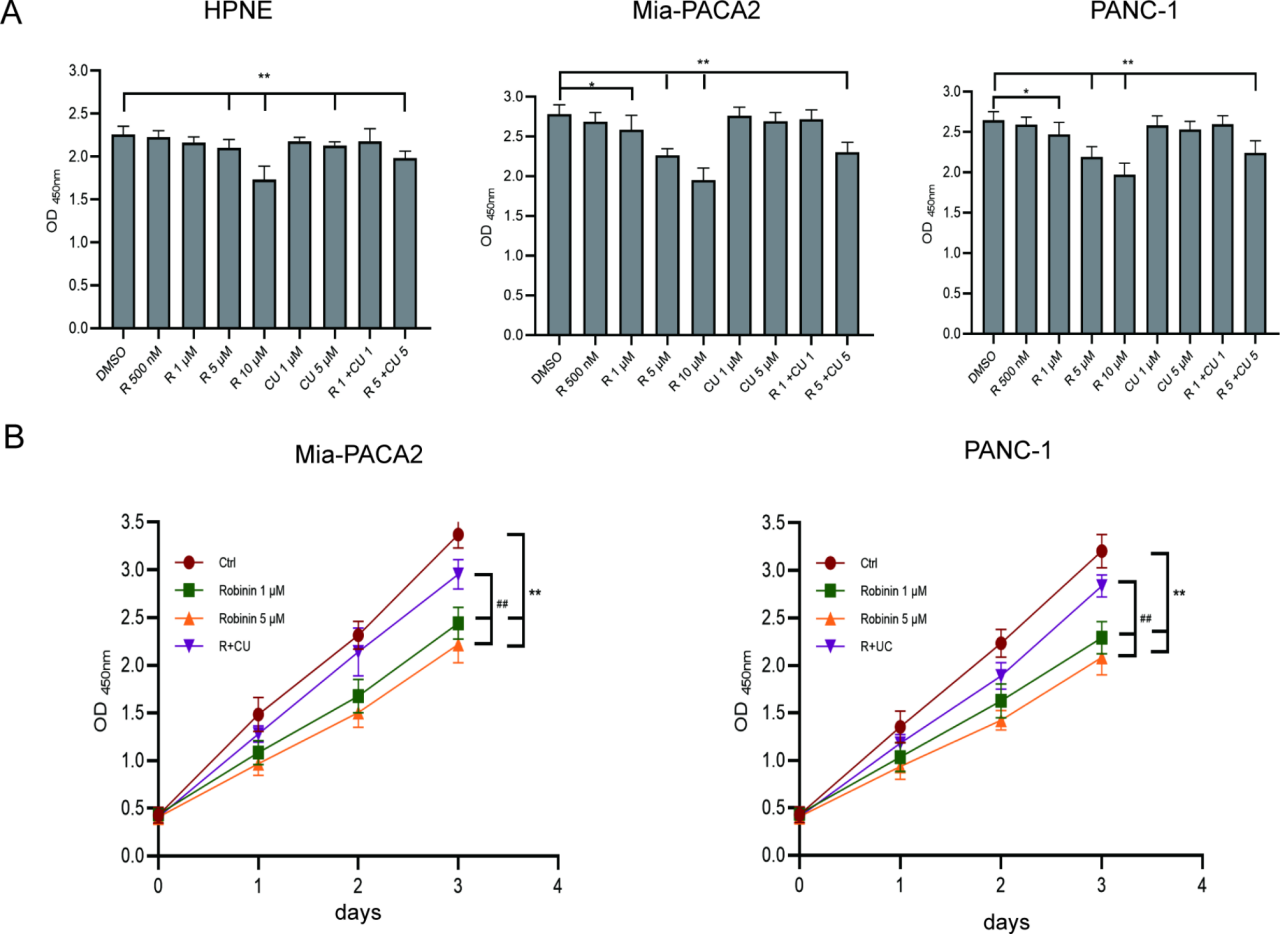
The effects of Robinin and CU-T12-9 on HPNE, Mia-PACA2 and PANC-1 viability and proliferation. **(A)** HPNE, Mia-PACA2 and PANC-1 were exposed to different concentrations of Robinin (500 nM, 1 µM, 5 µM or 10 µM) and CU-T12-9 (1 µM or 5 µM) for 24 h. **(B)** Proliferation curve of Mia-PACA2 and PANC-1 exposed to control medium in presence with Robinin (1 µM/ml or 5 µM/ml) and Robinin co-cultivated with CU-T12-9 for 24, 48 and 72 h. Data are presented as the means ± standard deviation of 3 independent repeats. *P < 0.05, **P < 0.01, vs. the control group, #*P* < 0.05, ##*P* < 0.01, vs. the Robinin co-cultivated with CU-T12-9 group by one-way ANOVA followed by LSD test

It was observed that Robinin (> 5 µM/mL) reduced cell viability significantly in Mia-PACA2 and PANC-1 compared that of the untreated groups (**P < 0.01, Fig. 3A). A slight cell toxicity in Mia-PACA2 was found in presence with Robinin (1 µM /ml) compared to the untreated group (450 nM OD values, Ctrl = 2.78 ± 0.16, Robinin 1 µM = 2.58 ± 0.18, *P = 0.019; Fig. 3A). A slight cell toxicity in PANC-1 was found in presence with Robinin (1 µM /ml) compared to the untreated group (450 nM OD values, Ctrl = 2.65 ± 0.11, Robinin 1 µM = 2.47 ± 0.15, *P = 0.029; Fig. 3A). No significant cell toxicity was observed in presence with CU-T12-9 (< 5 µM/ml, *P* > 0.01, Fig. 3A).

In HPNE, no cell toxicity was observed in presence with Robinin (1 µM/ml) compared to the untreated group (450 nM OD values, Ctrl = 2.26 ± 0.10, Robinin 1 µM = 2.16 ± 0.06, *P* = 0.144; Fig. 3A). Robinin o-cultivated with 1 µM/ml CU-T12-9 also showed no toxicity in HPNE (2.26 ± 0.10 vs. 2.18 ± 0.15, *P* = 0.220, Fig. 3B). Based on the toxicity results, 1 μm Robinin and co-cultivated with 1 μm CU-T12-9 were selected as the concentration for subsequent experiments.

Proliferation assay verified that Mia-PACA2 proliferation significantly diminished in the presence of Robinin of 1 µM/ml treatment for 72 h (3.37 ± 0.14 vs. 2.44 ± 0.17, **P < 0.01, Fig. 3B), but CU-T12-9 can partially reversed Robinin’s inhibition on Mia-PACA2 (2.95 ± 0.15 vs. 2.44 ± 0.17, ##*P* < 0.01, Fig. 3B). Similarly, 1 µM/ml Robinin strongly attenuated the proliferation of PANC-1 (3.20 ± 0.18 vs. 2.29 ± 0.17, **P < 0.01, Fig. 3B). Co-cultivated with CU-T12-9 also partly attenuate the inhibition of Robinin in PANC-1 (2.84 ± 0.12 vs. 2.29 ± 0.17, ##*P* < 0.01, Fig. 3B). Similar results were also found in the treatment of 5 µM/ml Robinin on Mia-PACA2 and PANC-1.

### Robinin inhibits cell migration of mia-PACA2 and PANC-1

**Fig. 4:**
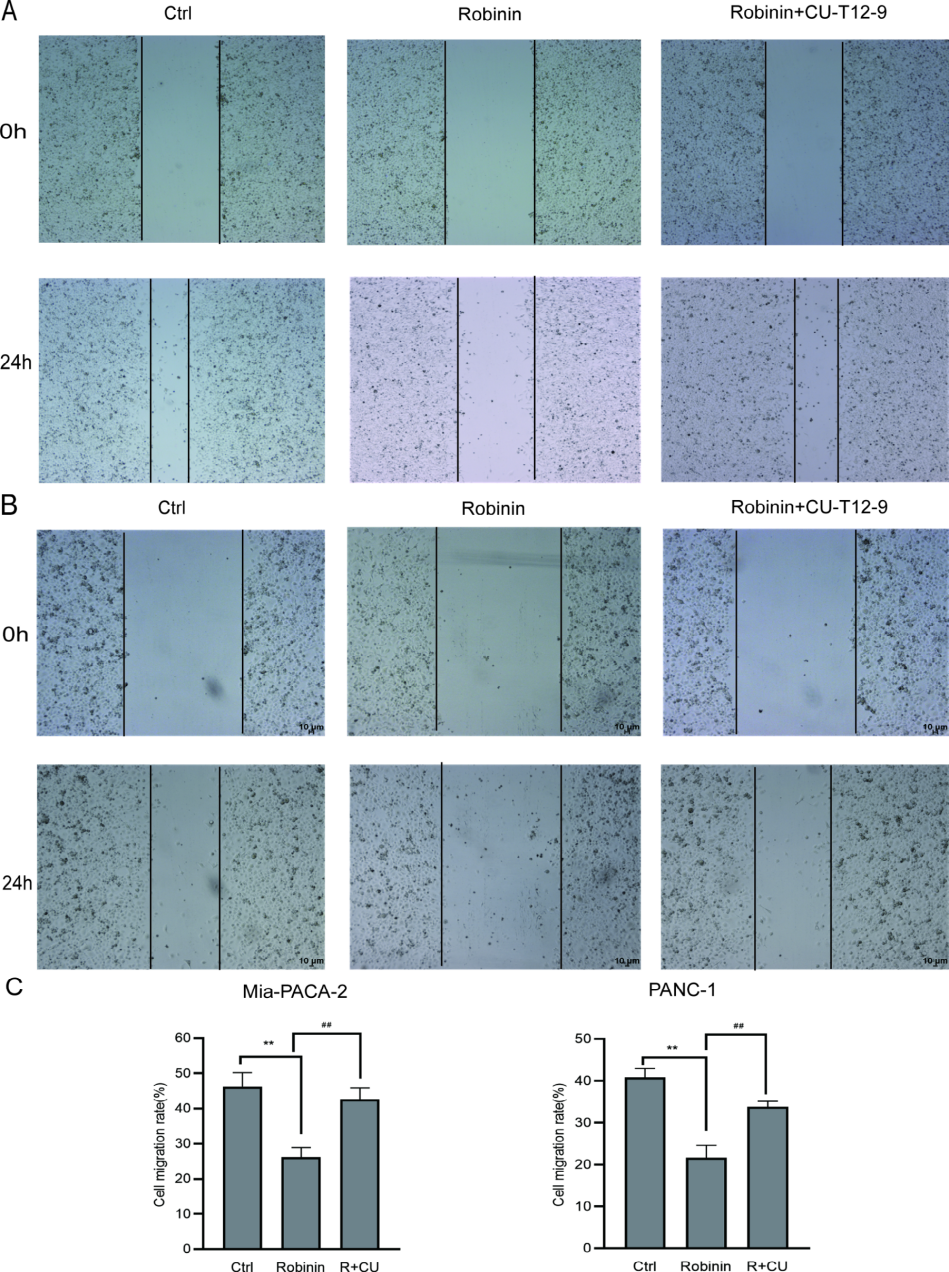
Robinin (1 µM/mL) inhibits cell migration in Mia-PACA2 and PANC-1. Cell migration was evaluated by wound‑healing assay. **(A)** Representative images of the different treatment groups in Mia-PACA2 at 24 h after scratch. **(B)** Representative images of different treatment groups in PANC-1 at 24 h after scratch. **(C)** Quantitative analyses of denuded area percentages (denuded area at the specified time point/denuded area at 0 h) after different treatments at different times in Mia-PACA-2 and PANC-1. Data are presented as the means ± standard deviation of 3 independent repeats. *P < 0.05, **P < 0.01, vs. the control group, #*P* < 0.05, ##*P* < 0.01, vs. the Robinin co-cultivated with CU-T12-9 group by one-way ANOVA followed by LSD test. Original magnification, x4

Wound healing assay was conducted to evaluate the migratory activity of the cells in vitro. Robinin obviously attenuated the migration of Mia-PACA2 and PANC-1, but in the presence of CU-T12-9 can significantly reverse the effect of Robinin. Treated with Robinin for 24 h, the cell migration area of Mia-PACA2 was about 26.20 ± 2.74%, much narrower than the area of ctrl group (46.27 ± 3.89%, **P < 0.01, Fig. 4A), while this effect was reversed by Robinin co-cultivated with CU-T12-9 (42.61 ± 3.32%, ##*P* = 0.01, Fig. 4A). Treated with Robinin for 24 h, the cell migration area of PANC-1 was about 21.64 ± 3.00%, much narrower than the area of ctrl group (40.86 ± 2.15%, **P < 0.01, Fig. 4B), while this effect was reversed by Robinin co-cultivated with CU-T12-9 (33.84 ± 1.29%, ##*P* = 0.01, Fig. 4B).

### Robinin attenuated EMT and inflammation of mia-PACA2 and PANC-1

**Fig. 5:**
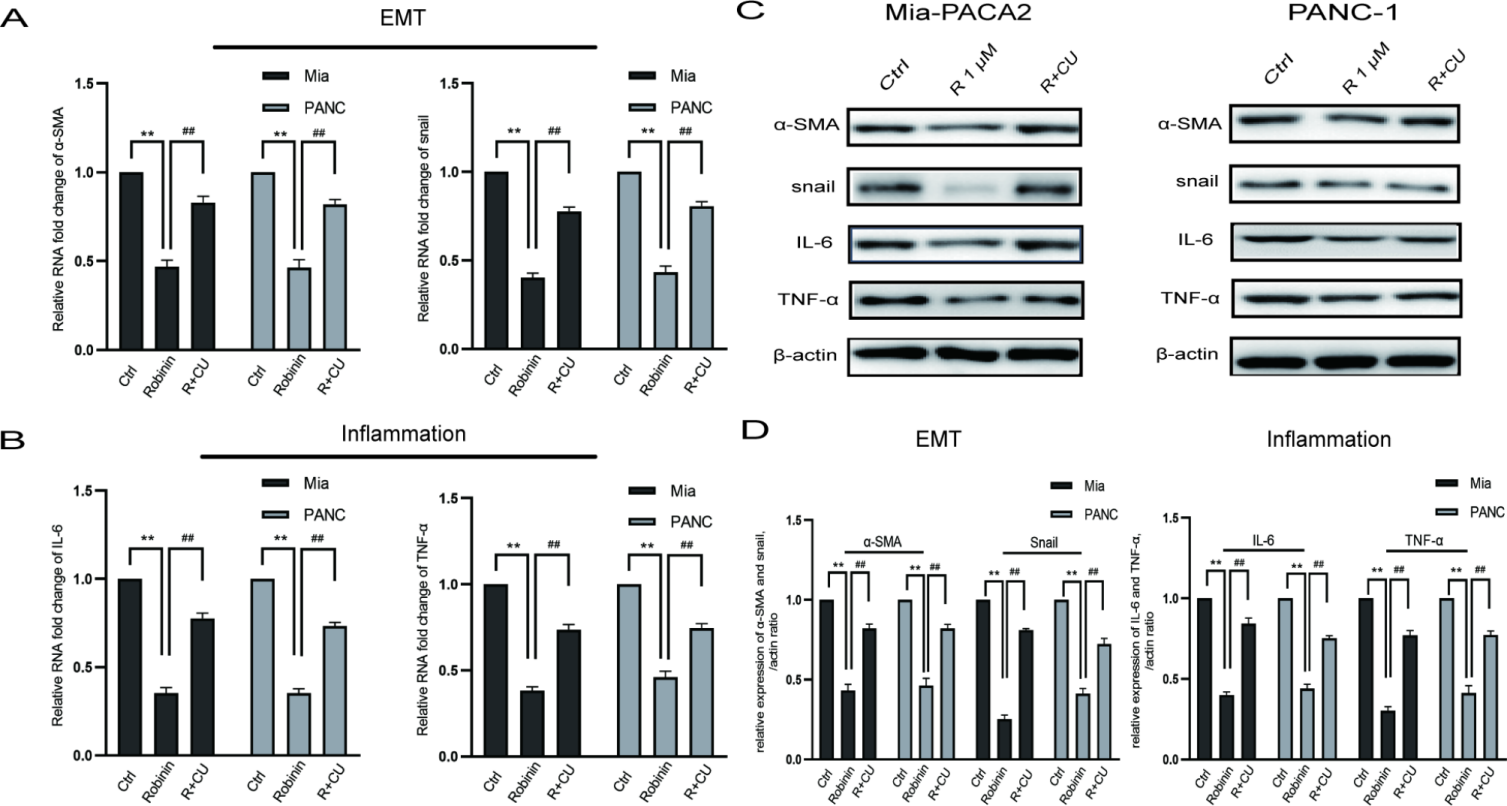
Robinin (1 µM/mL) alleviated EMT and inflammation in Mia-PACA2 and PANC-1. **(A)** qRT-PCR of α-SMA and snail expression in Mia-PACA2 and PANC-1. **(B)** qRT-PCR of IL-6 and TNF-α expression in Mia-PACA2 and PANC-1. **(C-D)** Protein levels of EMT and inflammation in Mia-PACA2 and PANC-1 were analyzed by western blot analysis and normalized to β-actin expression. *P* < 0.05, **P < 0.01, vs. the control group, #*P* < 0.05, ##*P* < 0.01, vs. the Robinin co-cultivated with CU-T12-9 group. Measurement data were expressed as mean ± standard derivation; data between PC cell and HPNE were compared by t-test, and measurement data among multiple groups were analyzed by one-way analysis of variance with Tukey’s post hoc test. The experiment was repeated three times

The typical markers of EMT were examined, including snail and α-SMA. Robinin (1 µM/ml) largely attenuated snail and α-SMA expression in Mia-PACA2 and PANC-1, which certified that Robinin can obviously inhibit the conversion progress of EMT in pancreatic cancer (Fig. 5A). Furthermore, the typical markers of inflammation were examined, including IL-6 and TNF-α. Robinin (1 µM/ml) largely attenuated IL-6 and TNF-α expression in Mia-PACA2 and PANC-1, which certified that Robinin can obviously inhibit the inflammation in pancreatic cancer (Fig. 5B).

### Robinin downregulated PI3K/AKT signal pathway in mia-PACA2 and PANC-1

**Fig. 6:**
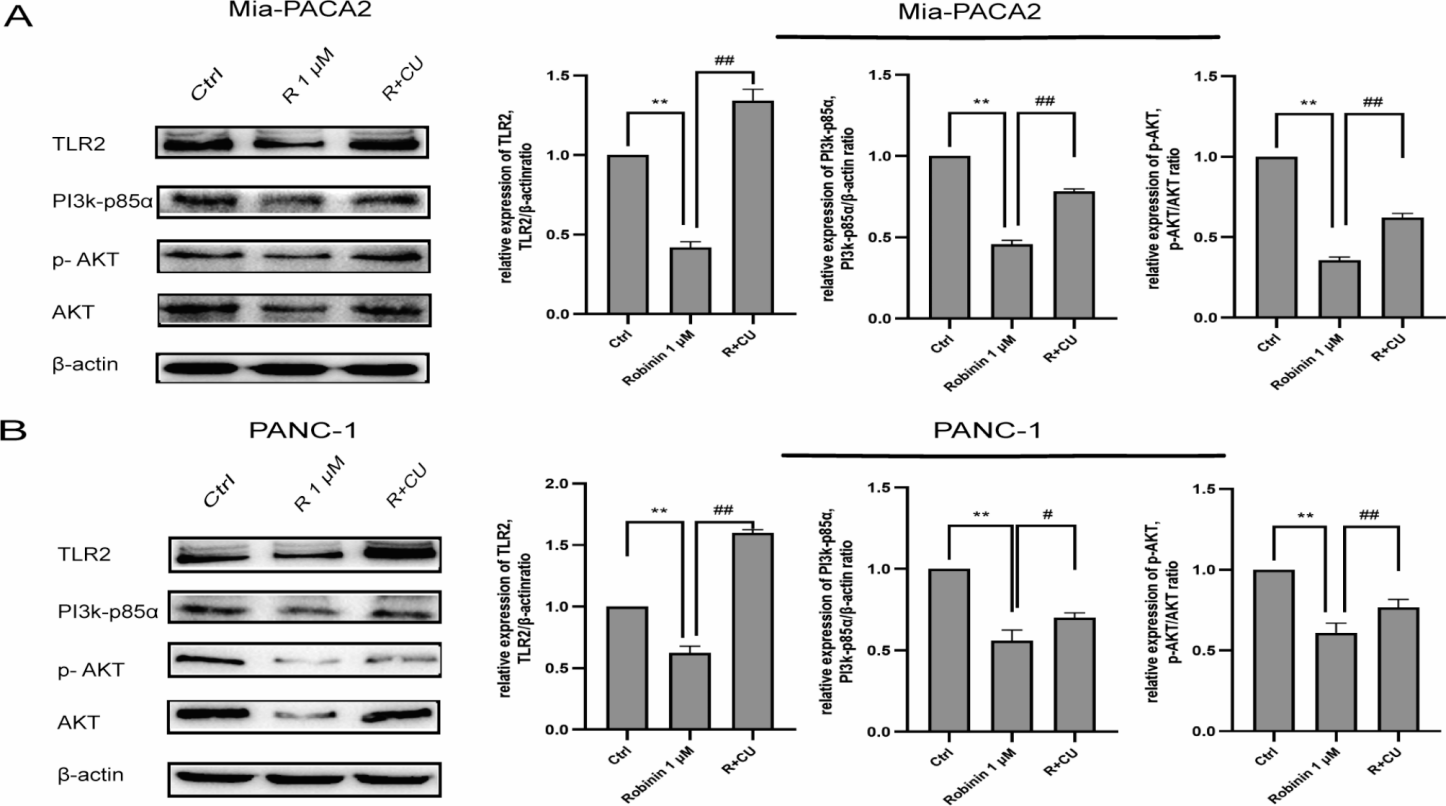
Robinin downregulated PI3k-AKT signal pathway via TLR2 in Mia-PACA2 and PANC-1. **(A)** The expression levels of TLR2, PI3k-p85α, and p-AKT in Mia-PACA-2 were detected by western blot analysis and normalized to β-actin and AKT, respectively. **(B)** The expression levels of TLR2, PI3k-p85α, and p-AKT in PANC-1 were detected by western blot analysis and normalized to β-actin and AKT, respectively. *P < 0.05, **P < 0.01, vs. the control group, #*P* < 0.05, ##*P* < 0.01, vs. the Robinin co-cultivated with CU-T12-9 group. Measurement data were expressed as mean ± standard derivation; data between PC cell and HPNE were compared by t-test, and measurement data among multiple groups were analyzed by one-way analysis of variance with Tukey’s post hoc test. The experiment was repeated three times

TLR2, PI3k-p85α, and p-AKT protein levels were detected, using western blot and normalized to β-actin and AKT proteins. In Mia-PACA2, western blot analysis showed that the expression levels of expressions of TLR2, PI3k-p85α, and p-AKT were significantly decreased by Robinin treatment in Mia-PACA2 (all **P < 0.01, Fig. 6A), whereas the Robinin effect was partly reversed by CU-T12-9 (##*P* < 0.01, Fig. 6A). Expressions of TLR2, PI3k-p85α, and p-AKT in PANC-1were also significantly decreased by Robinin treatment (all **P < 0.01, Fig. 6B), while these effects were partly reversed by CU-T12-9 (#*P* < 0.05, Fig. 6B). These results suggested that Robinin might play its biological function targeted on TLR2 then through regulating the PI3k-AKT signaling pathway.

### Pancreatic cancer development were inhibited in response to intraperitoneal injection of Robinin in pancreatic cancer model mice

**Fig. 7:**
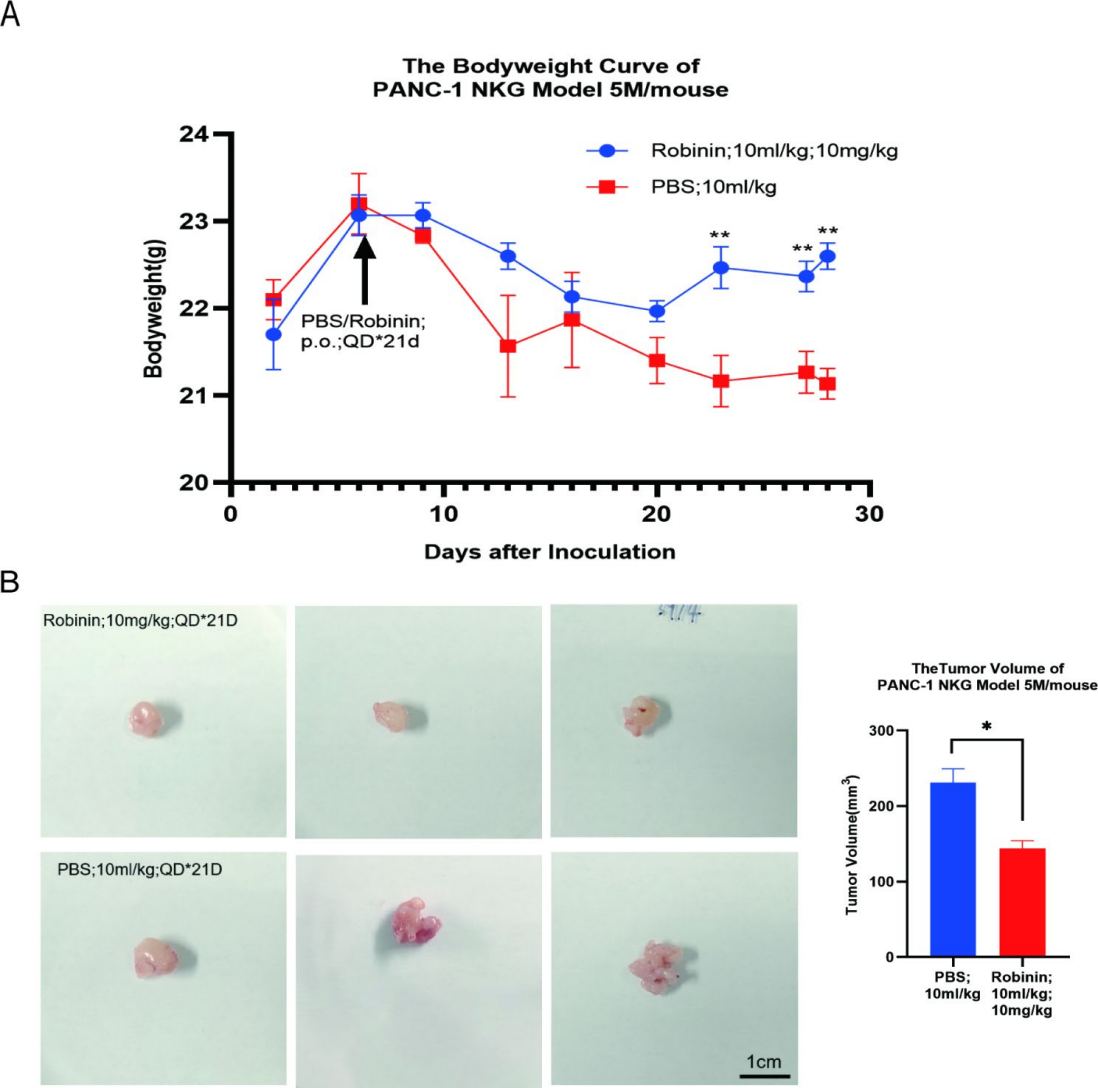
Robinin inhibited pancreatic cancer development in Panc-1 NKG mouse. **(A)** Weight loss was measured and analyzed between two groups. **(B)** Tumor volume were measured and analyzed between two groups. Scale bar, 1 cm. Data were presented as mean ± SD. *P < 0.05, **P < 0.01

**Fig. 8:**
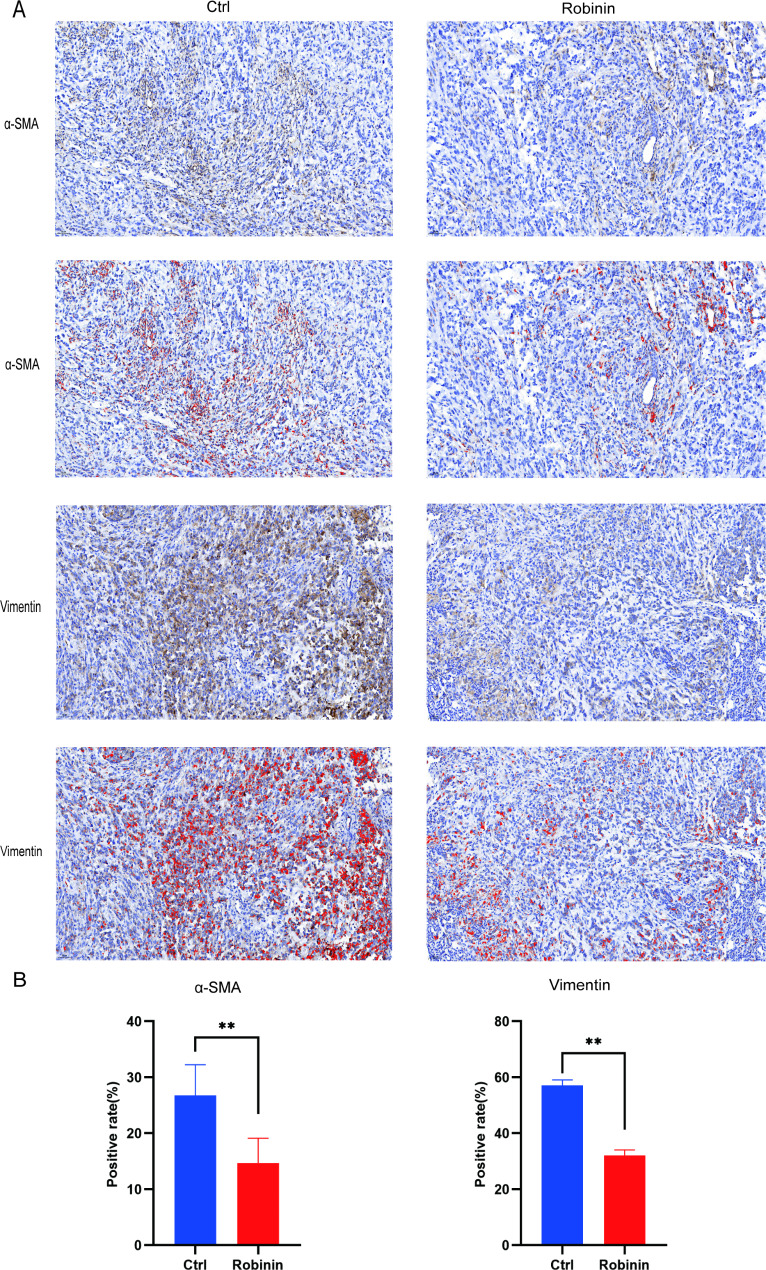
Robinin attenuated EMT and fibrosis in Panc-1 NKG mouse. **(A)** Immunohistochemistry was performed to detect vimentin and α-SMA expression and labeled with DAB (brownish red). The nuclei (blue) were labeled with hematoxylin. Scale bar, 200 mm. **(B)** Positive staining rate were counted and analyzed between two groups. Data were presented as mean ± SD. *P < 0.05, **P < 0.01

Consistent with our previous in vitro study, the weight loss (D9-D28 weight) of PANC-1 NKG mice treated with Robinin was obvious less than that of PBS induced PANC-1 NKG mice (Table 1; Fig. 7A), while the tumor volume of PANC-1 NKG mice with Robinin treated was also significant smaller than that of PANC-1 NKG mice (Table 1; Fig. 7B). The immunoreactivity of vimentin and α-SMA was analyzed for assessing tumor EMT, signs of malignant tumor growth (Figs. 8). The α-SMA immunoreactivity in the tumor tissue was mainly restricted to the interstitial tissue, while the vimentin immunoreactivity in the tumor tissue was largely distributed to the epithelium tissue. In the control NKG model mice, vimentin and α-SMA positive immunoreactivity were 26.78 ± 5.48 and 57.16 ± 3.36 (Fig. 8B). In the case of Robinin treated NKG model mice, vimentin positive immunoreactivity was 32.01 ± 3.48 which showed significance difference with control group (*P* = 0.001, Fig.8B). Similarly, α-SMA positive immunoreactivity of Robinin treated NKG model mice was 14.69 ± 4.42 which showed significance difference with control group (*P* = 0.041, Fig. 8B). Immunoreactivity of tumor tissues showed that vimentin and α-SMA were downregulated in Robinin treated mice, in which indicated that Robinin can partially inhibit development of pancreatic cancer.

## Discussion

Pancreatic cancer is a malignancy with a high mortality rate and increasing incidence and associated mortality, and even with treatment, its prognosis is extremely poor [15]. The occurrence and development of tumors is inseparable from the interaction of multiple factors and mutual promotion. Toll-like receptors are a group of receptors that play a key role in innate immune signaling and initiating inflammatory responses [16]. In recent years, emerging evidence has emerged that TLRs play an extremely important part in the progression of cancer. Studies have shown that among many TLR subtypes, TLR2 is highly overexpressed in pancreatic cancer, and its expression is associated with cancer aggressiveness [17, 18]. According to previous literatures, the migration and invasion ability of epithelial tumors is enhanced, and EMT is an important biological process for epithelial cell-derived malignant tumor cells to acquire migration and invasion ability. Changes in the expression of epithelial and mesenchymal genes during EMT are regulated by multiple families of transcription factors, including SNAI1/Snail, ZEB1/ZEB2, and basic helix-loop-helix transcription factors [19]. Studies have confirmed that inflammatory factors can promote the growth of tumors and play an important role in the process of tumor cell migration, invasion and metastasis. Our results show significant upregulation of expression levels of TLR2, Snail and α-SMA, and symptomatic markers (IL-6 and TNF-α).

Robinin which is found in plants of the genus *Astragalus* has been reported to have explicit anti-inflammatory, pain relieving effects, however, research on its anti-tumor effect is still limited [20]. Robinin’s anti-inflammatory effects has been confirmed by several studies. Eom et al. [21]have reported that Robinin can decrease the expression of several inflammation cytokines, such as IL-6, TNF-α, and cyclooxygenase-2 (COX-2) in inflammatory macrophages. In another adjuvant arthritis survey, Robinin has been applied combined with Methotrexate to strengthen the anti-inflammation effect through decreasing IL-17 in blood plasma [20]. However, only one manuscript focused on anti-tumor effect of Robinin on breast cancer has been found (Simultaneous determination of the flavonoids robinin and kaempferol in human breast cancer cells by liquid chromatography-tandem mass spectrometry) [22]. Thus, Robinin’s role in pancreatic cancer has not been reported yet. Our study shows that Robinin, as an inhibitor of TLR2, significantly inhibits the proliferation and migration of pancreatic cancer cells, which is reversed by the combination of CU-T12-9 and Robinin. At the same time, we also observed that Robinin can inhibit the expression of inflammatory factors and EMT in pancreatic cancer cells. Therefore, we demonstrated that Robinin possess anti-cancer biological function in PC perhaps by regulating the tumor immune microenvironment. The results of our research has potent clinical application value in pancreatic cancer treatment.

Interference of signaling pathways in human cancers provides a new area of cancer treatment [23]. The PI3K-AKT pathway, an intracellular signaling pathway, is often dysregulated in human cancers and has been shown to be central to mediating various cellular processes, including cell proliferation, apoptosis, and cell migration [24–26]. Studies have shown that the PI3K/Akt signaling pathway is closely related to the regulation of CircNDST1 in the proliferation and invasion of papillary thyroid cancer [27]. Guo et al. [28]suggest that ROR2/PI3K/Akt regulatory network may help promote breast cancer progression. Furthermore, increasing research into this pathway favors effective drug development and cancer treatment [29]. Therefore, the expression of ATK may reflect the proliferative capacity of the tumor, and its overactivation plays a crucial role in the development and development of cancer. We then explored the relationship between Robinin, TLR2, and the PI3k/ATK pathway. We observed that TLR2 protein expression was significantly inhibited and the PI3k/ATK pathway was activated after Robinin treatment, leading to proliferation and migration of pancreatic cancer cells. However, when Robinin and CU-T12-9 were co-treated, the above effects were successfully reversed by CU-T12-9.

Our study demonstrated that Robinin plays a positive role in inhibiting pancreatic cancer cell proliferation and migration. Furthermore, EMT and inflammation were also attenuated by Robinin treatment. Considering TLR is the most classical immunomodulatory receptor, the agents targeted on TLR may obtain effective applications in regulating TME of pancreatic cancer. In our study, Robinin appears to be a good candidate for inhibition of pancreatic cancer, namely anti-proliferation, anti-migration, and anti-EMT, which indicates Robinin’s potential value in the future clinical treatment of pancreatic cancer.

It should be stated that there are some limitations in the present study. In vitro assays are not ample to assess the anti-tumor effect of Robinin in vivo. Thus, it cannot be extrapolated directly in clinical situations. So, the anti-tumor effects of Robinin warrants investigation in animal model further.

## Conclusion

In summary, Robinin inhibited cell migration, proliferation, and attenuated EMT perhaps *via* regulating inflammatory microenvironment in pancreatic cancer cells through targeted on TLR2- PI3k-AKT signaling pathway. Our data strongly supports that Robinin may be a potential adjuvant therapy for pancreatic cancer.

### Electronic supplementary material

Below is the link to the electronic supplementary material.

**Supplementary Material 1:** Original image of WB-1

**Supplementary Material 2:** Original image of WB-2

**Supplementary Material 3:** Original image of WB-3

**Supplementary Material 4:** Original image of WB-4

**Supplementary Material 5:** Original image of WB-5

**Supplementary Material 6:** Original image of WB-6

**Supplementary Material 7:** Original image of WB-7

## Data Availability

The datasets used and/or analyzed during the present study are available from the authors on reasonable request.

## Abbreviations

BSA: Bovine serum albumin
CCK-8: Cell counting kit‑8
DMEM: Dulbecco’s modified Eagle medium
EMT: Epithelial‑mesenchymal transition
FBS: Fetal bovine serum
IL-6: Interleukin- 6
TNF-α: Tumor necrosis factor-α
TLR: Toll like receptor
α-SMA: α-smooth muscle actin
