# Supplementary figures and images for: Robinin inhibits pancreatic cancer cell proliferation, EMT and inflammation via regulating TLR2-PI3k-AKT signaling pathway
Source: Cancer Cell Int. 2023 Dec 18;23:328. doi: 10.1186/s12935-023-03167-3 (PMC10726507; doi:10.1186/s12935-023-03167-3)

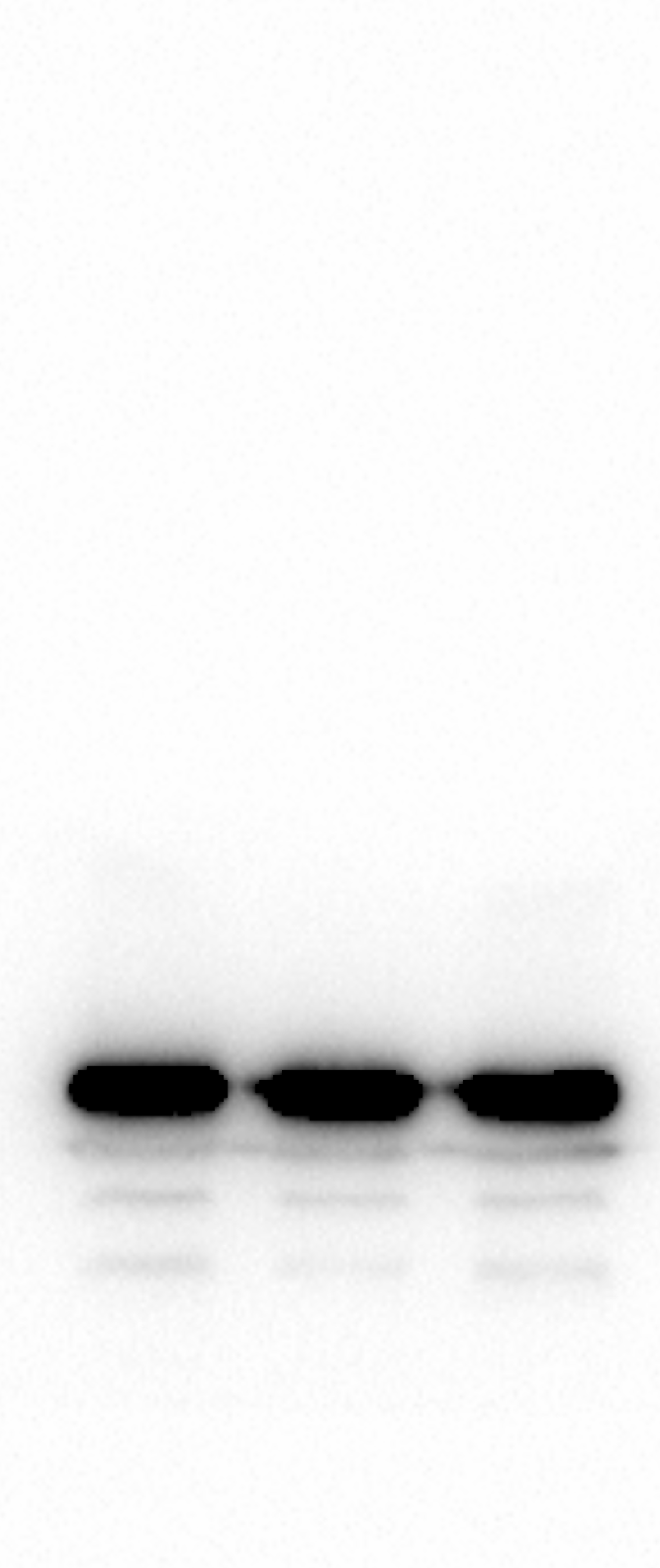

Supplement: Supplementary file 1 — Supplementary Material 1: Original image of WB-1 [file 12935_2023_3167_MOESM1_ESM.tif]

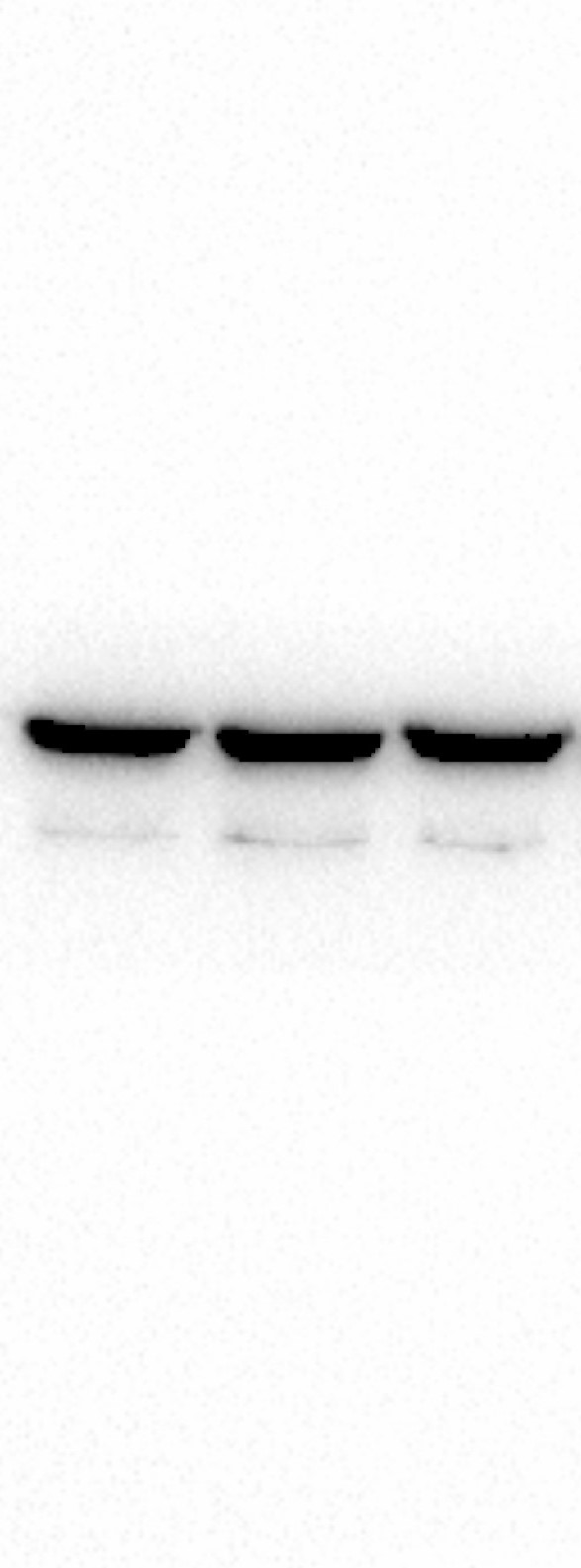

Supplement: Supplementary file 2 — Supplementary Material 2: Original image of WB-2 [file 12935_2023_3167_MOESM2_ESM.jpg]

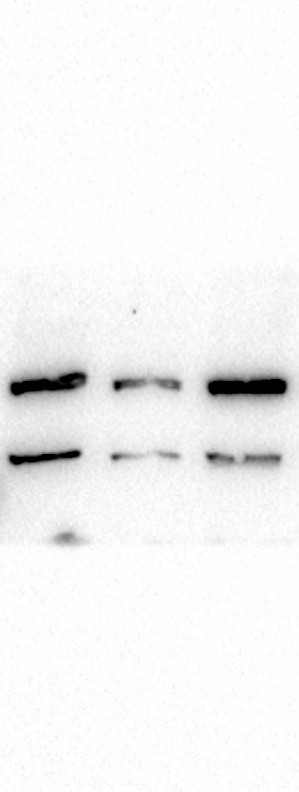

Supplement: Supplementary file 3 — Supplementary Material 3: Original image of WB-3 [file 12935_2023_3167_MOESM3_ESM.tif]

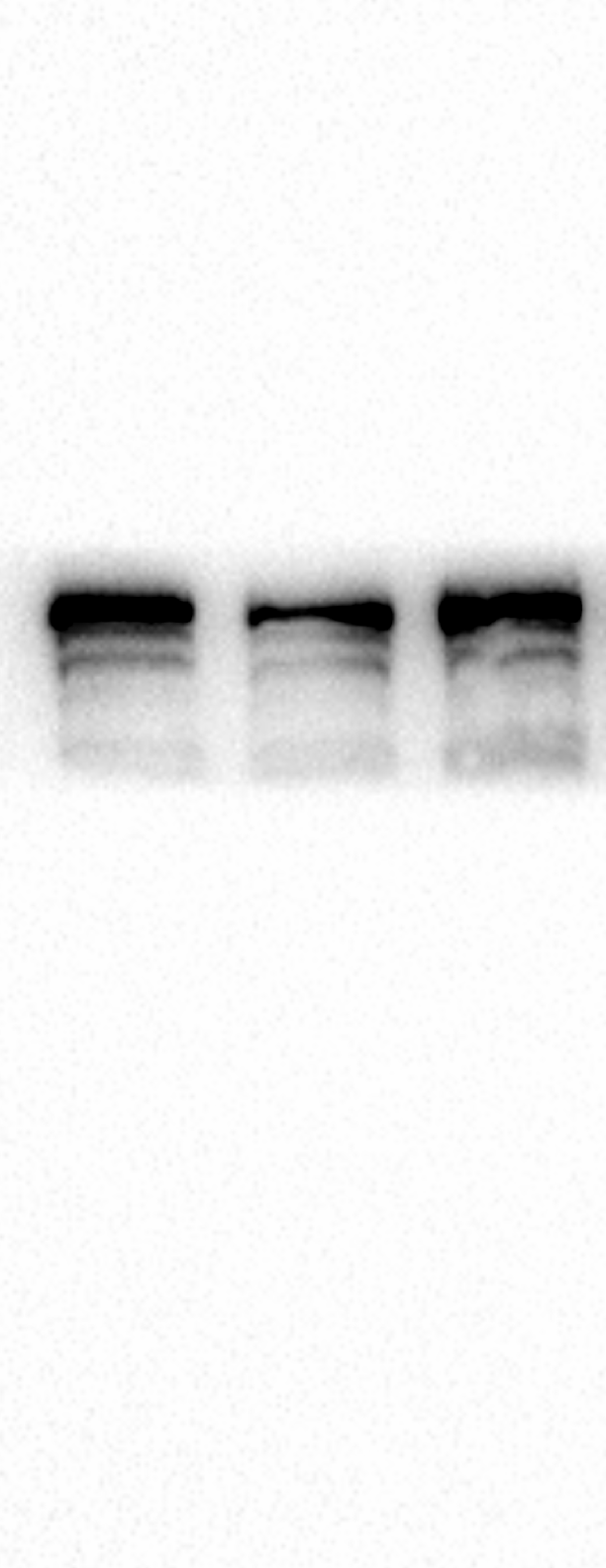

Supplement: Supplementary file 4 — Supplementary Material 4: Original image of WB-4 [file 12935_2023_3167_MOESM4_ESM.tif]

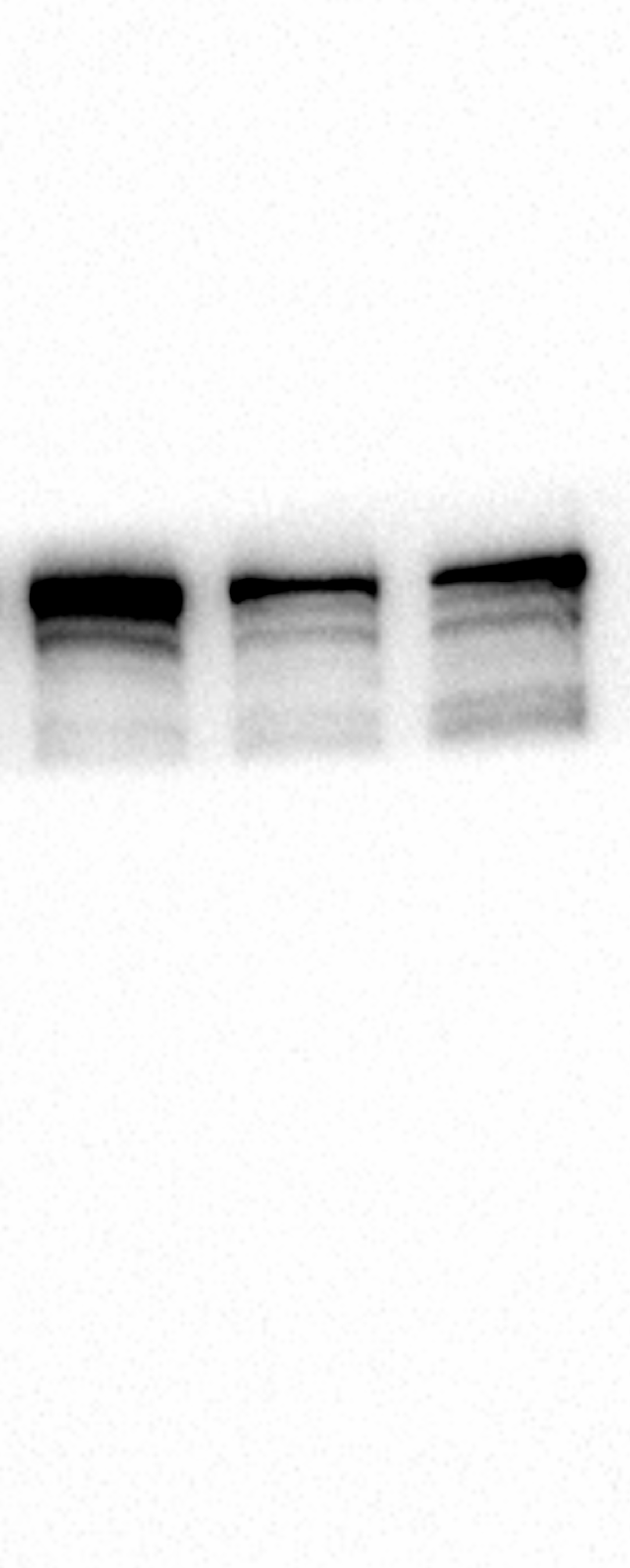

Supplement: Supplementary file 5 — Supplementary Material 5: Original image of WB-5 [file 12935_2023_3167_MOESM5_ESM.tif]

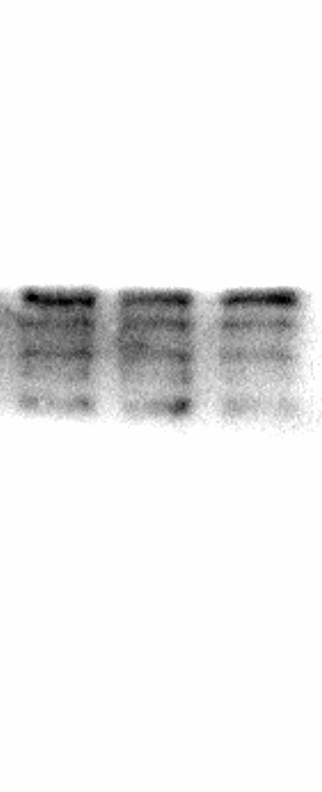

Supplement: Supplementary file 6 — Supplementary Material 6: Original image of WB-6 [file 12935_2023_3167_MOESM6_ESM.tif]

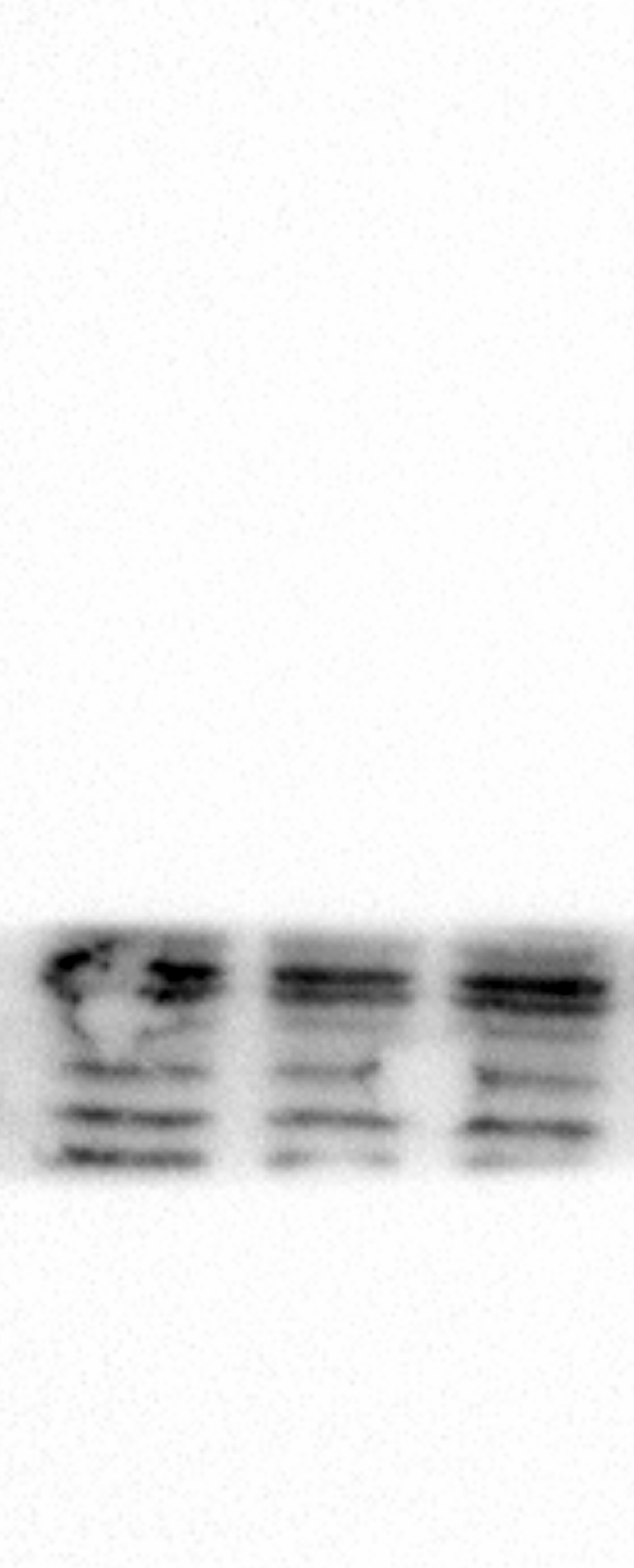

Supplement: Supplementary file 7 — Supplementary Material 7: Original image of WB-7 [file 12935_2023_3167_MOESM7_ESM.tif]
